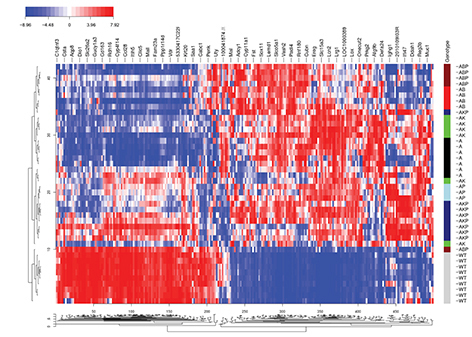# Analysis of GEMMs helps unravel genetic heterogeneity in colorectal cancer

**Published:** 2014-06

The majority of cases of colorectal cancer (CRC), one of the most commonly diagnosed cancers worldwide, arise sporadically by the acquisition of tumorigenic somatic alterations. Sporadic CRC is difficult to mimic in preclinical animal models because of its heterogeneous nature, which has contributed to the current lack of effective CRC therapies. Genetically engineered mouse models (GEMMs) have been widely used to explore cancer biology and establish targeted cancer therapies. In this study, Eric Martin and colleagues at Pfizer used a unique collection of GEMMs harbouring colon-specific mutations that have been commonly observed in CRC to help resolve the genetic heterogeneity. Primary tumour material isolated from the GEMMs was used to generate gene expression signatures, which were then applied to human CRC datasets to stratify cancer subsets based on genotype. The authors show that a signature based on a mutant *Kras* allele can distinguish carriers of the human *KRAS* mutation in the clinical samples and, furthermore, that this signature correlates with poor patient prognosis. Finally, they demonstrate that high expression of the GEMM Kras signature predicts sensitivity to a commonly used anticancer therapy in human CRC cell lines. This study supports the use of GEMMs as powerful preclinical tools for the study of molecular heterogeneity in CRC and for testing potential therapies. **Page 613**

**Figure:**